# Supplementary figures and images for: Efficacy and safety of tranexamic acid in intracranial haemorrhage: A meta-analysis
Source: PLoS One. 2023 Mar 31;18(3):e0282726. doi: 10.1371/journal.pone.0282726 (PMC10065302; doi:10.1371/journal.pone.0282726)

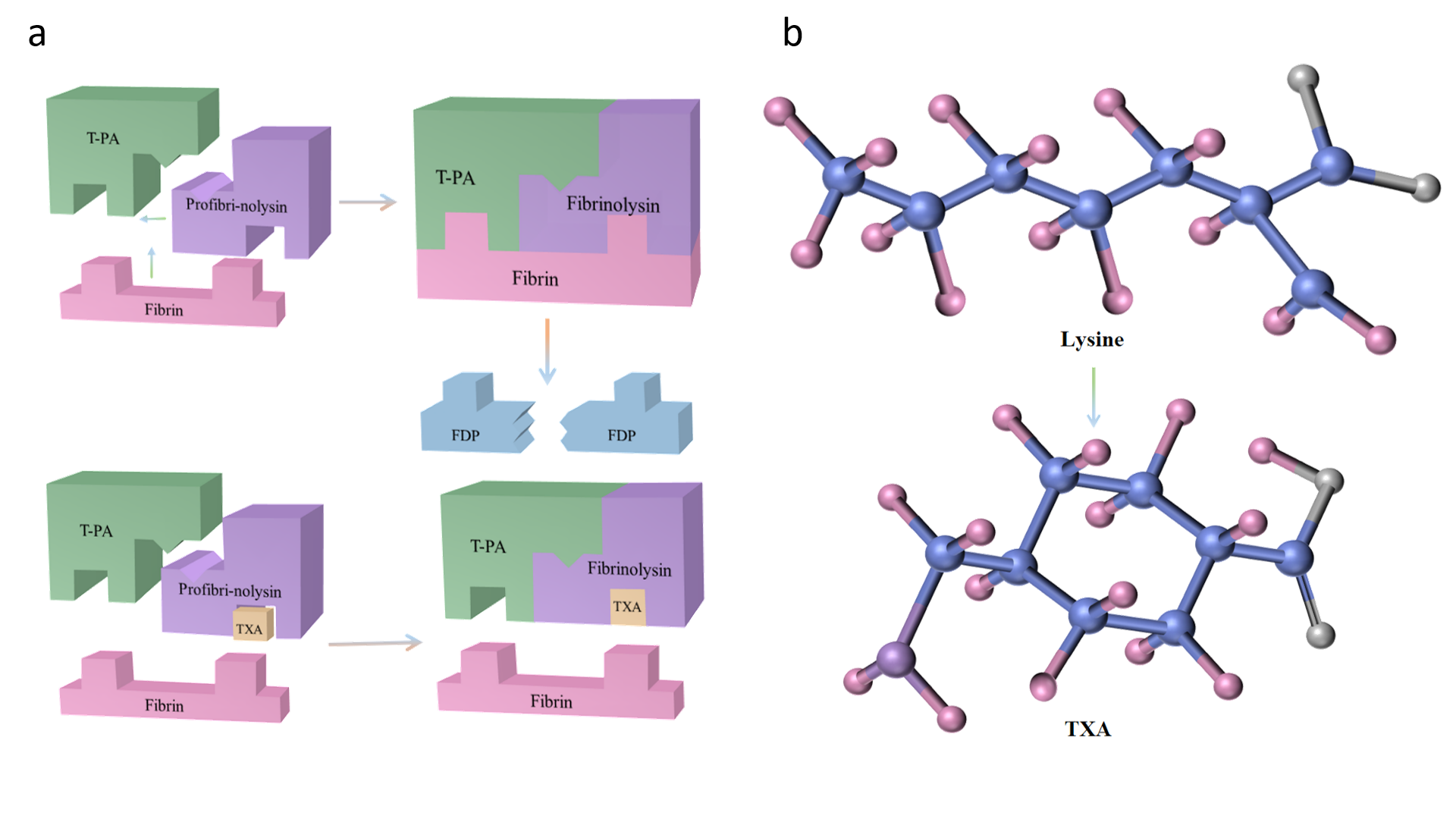

Supplement: S1 Graphical abstract — Schematic diagram of hemostatic mechanism of tranexamic acid (a) and chemical structure of tranexamic acid (b). (TIF) [file pone.0282726.s002.tif]

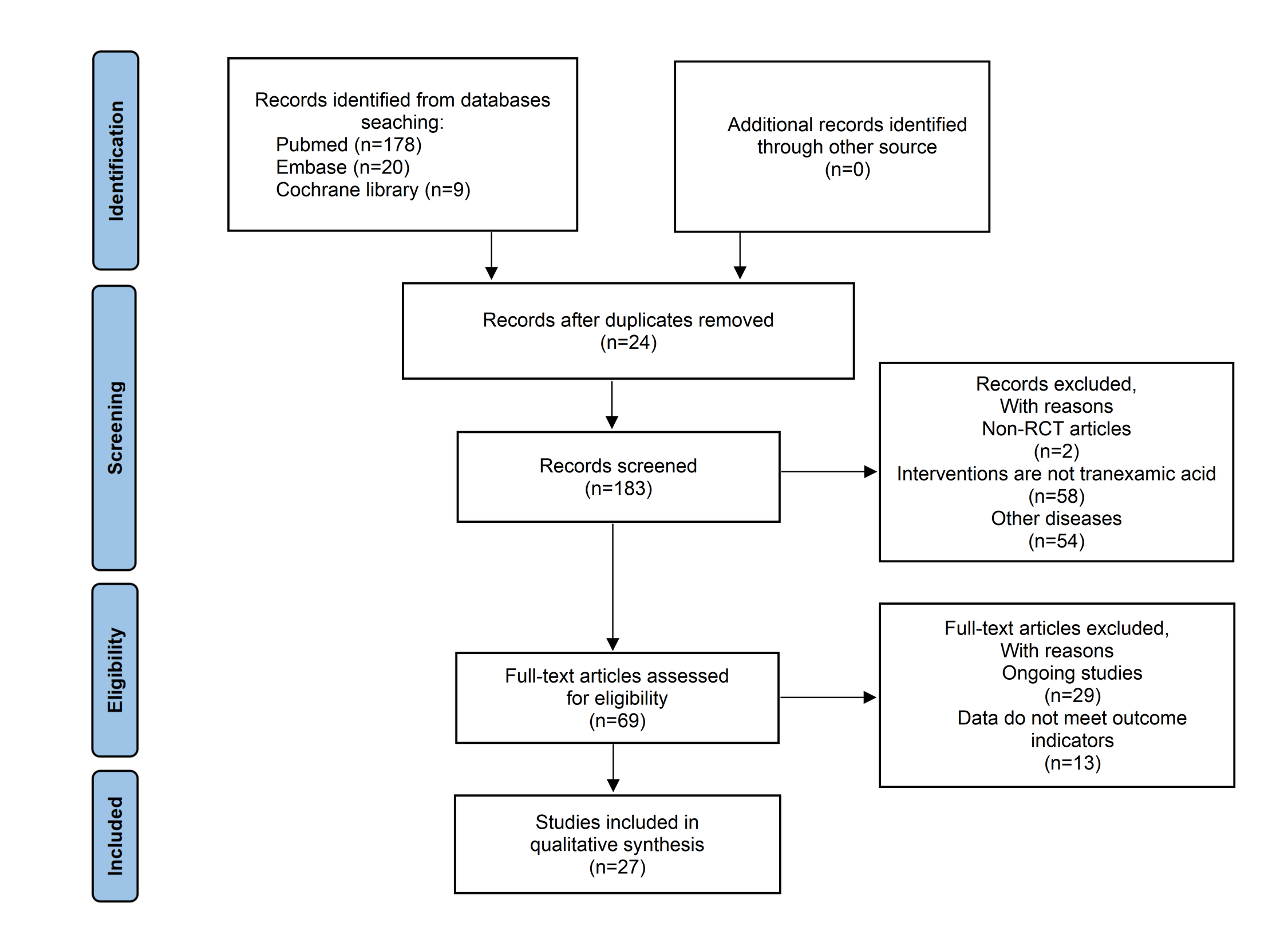

Supplement: S1 Fig — (TIF) [file pone.0282726.s003.tif]

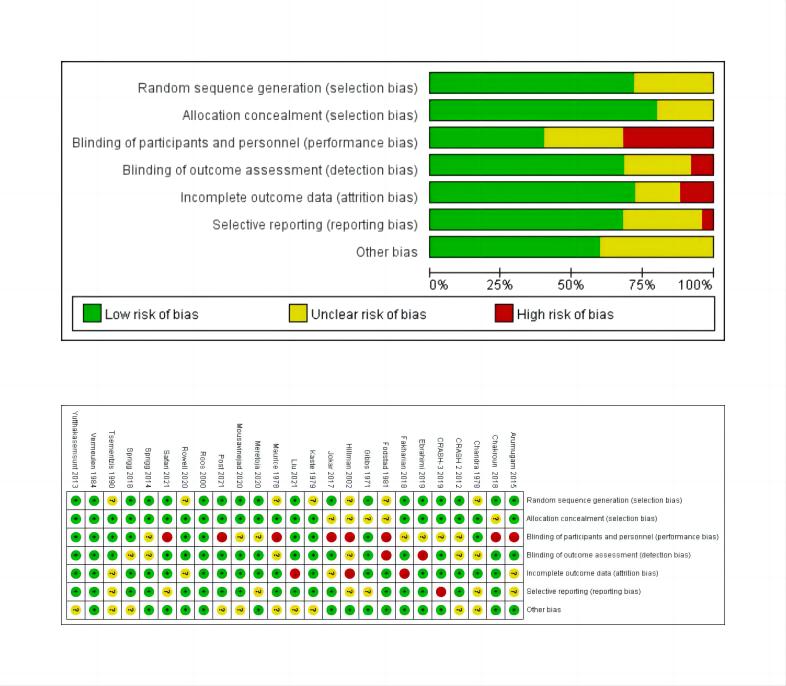

Supplement: S2 Fig — (a) The judgment of each bias risk item is expressed in percentage in all included studies. (b) Risk of bias summary. (JPG) [file pone.0282726.s004.jpg]
